# Infantile Umbilical Hernia—Its Causation, Symptoms, and Treatment

**Published:** 1898-05-21

**Authors:** W. McAdam Eccles

**Affiliations:** Assistant Surgeon to the City of London


### Medical Progress and Hospital Clinics.

[*The Editor will be glad to receive offers of co-operation and contributions from members of the profession. All letters should be addressed to* The Editor, at the Office, 28 & 29, Southampton Street, Strand, London, W.C.]

### INFANTILE UMBILICAL HERNIA—ITS CAUSATION, SYMPTOMS, AND TREATMENT.

By W. McAdam Eccles, M.S. (Lond.), F.R.C.S. (Eng.), Assistant Surgeon to the City of London Truss Society, to the West London Hospital, &c.

The term Infantile Umbilical Hernia is used to denote a protrusion at the umbilicus in an infant after the fall of the umbilical cord. Thus it is in reality an acquired hernia, and is wrongly styled congenital. It is of very frequent occurrence, and although it but seldom gives rise to dangerous symptoms, yet it is of sufficient importance to demand attention and treatment.

**Causation.**—It is not in any way dependent upon a congenital defect, but it is due to the gradual distension of the umbilical scar. An infant often within the first few months of life, either when nursed by the mother, or more frequently when it is hand-fed, develops dyspepsia. This is likely to lead to flatulent distension of the intestines, to be accompanied by a considerably increased intra-abdominal pressure and colicky pain. Here we have two very potent predisposing causes of hernial protrusions. The pressure tends to force the abdominal viscera through the weak spots of the abdominal wall; the colic, in addition, causes the child severe pain, with the result that it gives way to fits of crying which cannot be controlled. This naturally still further increases the tension within the abdomen. Therefore, I think that improper feeding has a very important share in the causation of infantile umbilical hernia. Children the subjects of this condition may be either fat or thin; when covered with adipose tissue the infant is often the victim of over-feeding, which has led first to the obese state and then to indigestion and its accompanying distress, but when the child is thin it usually suggests that there has been insufficient food or that digestion is at a very low ebb. In both states it will generally be seen that the abdomen is distended, and that it is markedly tympanitic everywhere on percussion.

**Symptoms and Signs.**—There is a more or less well-marked protrusion at the umbilicus, which very distinctly increases in size when the patient cries or otherwise strains. If there is much distension of the abdomen the swelling produced by the hernia may remain prominent, even when the child is lying on its back, but often the hernia disappears to some extent when the dorsal position is assumed. The contents of the sac are, however, almost always reducible, for an irreducible infantile umbilical hernia is decidedly rare. The reduction is affected with a characteristic gurgle of intestine, and the feel of a solid body slipping away from the fingers.

**Prognosis.**—If left alone these protrusions tend to increase in size so long as the infant is the subject of dyspepsia, that is until it reaches the age of about twelve months or more. After this, since the abdominal distension usually passes off, the swelling gradually becomes less and less, the hernial ring at the umbilicus at the same time diminishes in calibre, and ultimately becomes completely closed, with a permanent cure of the affection.

Such a spontaneous disappearance is the rule, for it is quite the exception to see an umbilical hernia in a patient about the age of puberty, which has persisted from the first year of life. Moreover, it is extremely doubtful whether a protrusion at the umbilicus in early infancy which disappears as childhood advances, is ever a predisposing cause of an acquired umbilical hernia. It is very rare for an infantile umbilical hernia to become strangulated, and if it did reduction would probably be easily effected by the taxis.

**Treatment.**—The treatment of these protrusions is generally quite simple. As there is a natural tendency to a spontaneous cure, all that is needed is to aid Nature's efforts to bring this about. First of all attention must be paid to the question of a proper diet for the child, for if this is not done, all efforts apart from it will tend to be useless. In addition to this some form of apparatus should be applied, in order that the viscera may be prevented from protruding. In this way the ring will quickly begin to contract. The best means to restrain the hernia is either to adjust a pad and strapping, or a spring truss. The former is useful in very young infants, the latter in those who are older. It is often stated that a penny wrapped in lint makes a good pad, but there is one serious objection to this form of appliance, in that by the action of the moisture of the body the copper of the coin tends to become corroded, and thus is likely to produce irritation and excoriation. If a silver coin is used, this effect is not so often produced. However, the best material to form the basis of the pad is undoubtedly a round piece of gutta percha, such as is used to mould splints out of. The size of this should be about that of a crown piece. It is to be wrapped in two folds of lint, which have to be daily changed. This pad is kept in position by means of a strip of Leslie's strapping, about two inches wide.

The adjustment of this is a matter of some importance. It is best to have the strapping long enough to reach rather more than completely round the child's body. The patient is to be laid on the piece of strapping, which is then brought to the front of the abdomen and so over the pad. In this way there is but little possibility of the child in its movements causing the apparatus to become displaced. It is necessary to change the pad at least once in a day, otherwise irritation is sure to ensue. When doing this the strapping is only loosened from the front of the pad and left untouched behind. The whole should be removed weekly. A small umbilical truss of the same construction and shape as that used for an adult is very satisfactory in a child who does not tend to render it sodden with urine.

It is extremely important to remember that any attempt to press a conical or other shaped projection from a pad into the umbilical ring is absolutely a wrong method of treatment. For if this were to be really accomplished it would tend to keep the hernial opening perpetually patent and thus effectively prevent a cure. Fortunately, if such an appliance is adjusted, it is almost invariably displaced by the child's movements so as to lie anywhere but in the ring itself. Seeing that there is such a marked tendency for this form of hernia to become spontaneously cured, it must be but seldom that a surgeon will find it necessary to recommend any kind of operative treatment.

